# Comparing the Efficacy of Povidone-Iodine Versus Normal Saline in Laparotomy Wound Irrigation to Prevent Surgical Site Infections: A Meta-Analysis

**DOI:** 10.7759/cureus.49853

**Published:** 2023-12-02

**Authors:** Christie Swaminathan, Wei H Toh, Ahmed Mohamed, Hussameldin M Nour, Mirza Baig, Muhammad Sajid

**Affiliations:** 1 Department of Digestive Disease and General Surgery, Royal Sussex County Hospital, Brighton, GBR; 2 Department of Pediatric Medicine, Tameside General Hospital, Manchester, GBR; 3 Department of General Surgery, Furness General Hospital, Furness, GBR; 4 Department of Surgery, Worthing Hospital, Worthing, GBR

**Keywords:** meta-analysis, wound irrigation, surgical site infection, laparotomy, povidone-iodine

## Abstract

Surgical site infections (SSIs) are a known complication of laparotomies and intra-abdominal surgical operations leading to remarkable consequences on mortality, morbidity, and expenses. The study aims to assess the efficiency of irrigating laparotomy incision sites with povidone-iodine (PVI) or normal saline (NS) in diminishing the rate of SSIs in patients undergoing gastrointestinal operations for varying indications.

Randomized controlled trials (RCTs) highlighting the contribution of laparotomy wound irrigation with PVI in opposition to NS in patients planned for laparotomy addressing numerous gastrointestinal issues, and their role in reducing SSI risk were obtained via searching of standard electronic medical databases. The analysis was conducted by utilizing meta-analysis principles procured by statistical software RevMan version 5.3 (Cochrane Collaboration, London, UK).

The yield of medical databases exploration and inspection was 13 RCTs on 3816 patients who underwent laparotomy for different gastrointestinal operations. There were 1900 patients in the PVI group whereas 1916 patients received NS wound irrigations preceding closure of the laparotomy skin wound. In the random effects model analysis, the use of PVI for laparotomy wound irrigation was associated with the reduced risk (odds ratio = 0.54, 95% CI (0.30, 0.98), Z = 2.04, P = 0.04) of SSIs. Nevertheless, there was outstanding heterogeneity (Tau^2^ = 70; chi^2^ = 40.19, df = 12; P = 0.0001; I^2^ = 70%) among the included studies.

According to the comprehensive analysis outcomes, it has been clinically proven that the use of PVI is highly effective in reducing the occurrence of SSIs, as well as their subsequent implications.

## Introduction and background

Surgical site infections (SSIs) are surgical complications leading to profound hardship in postoperative surgical patients manifested as extended intravenous or oral antibiotics use, lengthy wound care and hospital stay, and repeated radiological and surgical interventions [1]. As a result, SSIs account for an additional financial burden on healthcare systems worldwide. Furthermore, SSIs can cause delays in returning to normal work and suboptimal health-related quality of life of patients resulting in secondary loss of finances and dependence on long-term social support. Intra-abdominal sepsis following colorectal surgery and small bowel resection surgery leading to stoma formation, which adds significant financial burden, is a prime example.

In the UK, in 2021-2022, the incidence of SSIs reported amounted to 7.9% and 7.3% following large bowel and small bowel surgery, respectively [2]. In the USA, an incidence of 0.5% to 3% of SSIs across all surgical specialties has been documented [3]. There has been inconsistent SSI reporting data from Europe, with SSI incidence fluctuating between 1.5% and 20%. Moreover, SSI incidence of 14.4% and 20.0% in middle and low-income countries, respectively, was noted [4]. The overburden to manage post-operative infective complications was gauged to be between 1.47 billion and 19.1 billion euros [5]. Comparatively, it has been estimated that the cost of SSI management in the NHS can be $700 million, whereas in the USA, it can be approximately $3.3 billion annually [6,7].

Reduction in the aforementioned financial burden is achievable by implementing several diverse, yet effective measures [8]. These measures to lessen the incidence of SSI have been suggested and can be subdivided into three categories: pre-operative, intra-operative, and post-operative measures [9]. Pre-operative use of prophylactic antibiotics and sterile wound drapes in a number of operations illustrated profitable improvement by reducing the SSI incidence [10,11]. Various irrigation solutions have been used intra-operatively, including contrasting concentrations of povidone-iodine (PVI) and saline that were proven effective in reducing the incidence of SSIs [12-14]. In the context of digestive system disease surgeries, intra-peritoneal lavage is often considered a rational step, performed following colorectal or small bowel resections with the intent to reduce the emergence of SSIs [15]. A noteworthy opposing opinion is that intra-abdominal lavage in the context of emergency trauma laparotomy may not be as effective in tapering down the incidence of SSIs [16]. Laparotomy wound irrigation by various solutions such as antibiotics solution, normal saline (NS) solution, water solution, PVI, and chlorhexidine solution have also been attributed to reducing the incidence of SSIs. Both PVI and NS for wound irrigation have been reported to be helpful in preventing SSIs, but there does not seem to be solid evidence in the literature to suggest that a single agent surpasses the other [17,18].

If the efficacy of PVI resembles that of NS, then NS would instinctively be the preferred wound irrigation in terms of cost-effectiveness. The objective of this study, which was presented as an abstract in the Annual Congress of the Association of Surgeons of Great Britain and Ireland on 17-19 May 2023, is to evaluate the role of laparotomy wound irrigation either by PVI or NS in patients undergoing gastrointestinal laparotomy and assess their role in reducing the incidence of SSIs.

It is noteworthy to mention that numerous healthcare organizations and institutes across the globe have been suggesting and promoting measures to reduce SSI incidence as they proved their burden.

## Review

Guidelines

This work has been reported under AMSTAR (Assessment of Multiple Systematic Reviews) and PRISMA (Preferred Reporting Items for Systematic Reviews and Meta-Analyses) guidelines and a PRISMA flowchart has been created and included in line with the PRISMA guidelines.

Literature exploration and data basis

**Figure 1:**
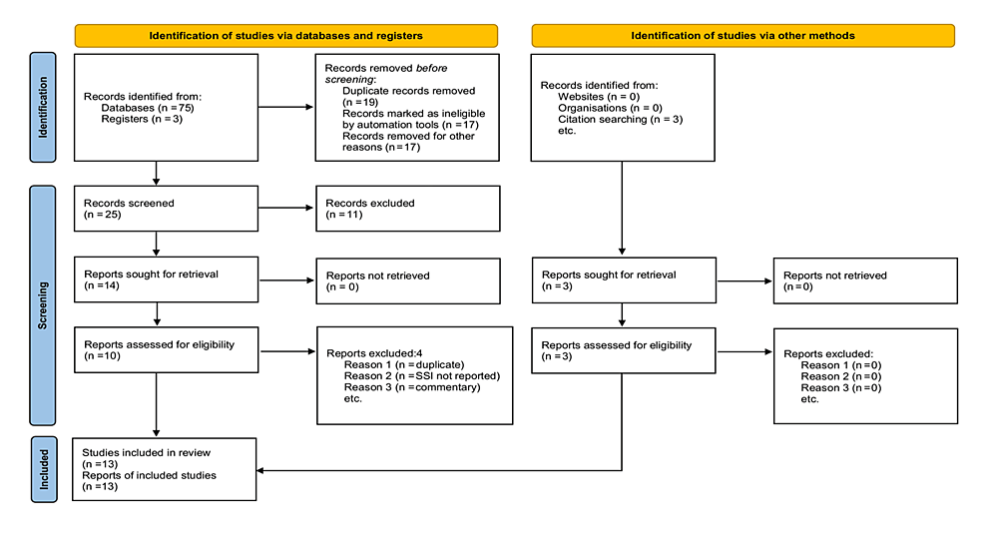
PRISMA flow chart showing literature search strategy and trial selection. PRISMA: Preferred Reporting Items for Systematic Reviews and Meta-Analyses.

The standard electronic databases such as MEDLINE, Embase, and Cochrane Library for all the published randomized controlled trials (RCTs) were navigated to locate the appropriate studies included in this meta-analysis. The Medical Subject Heading (MeSH) search terms (surgical site infection, SSI, laparotomy, wound irrigation, povidone-iodine wound wash, saline wound wash, large bowel resection, small bowel resection, etc.) given in the MEDLINE library related to the target objective were used to narrow down the relevant RCTs. In this search, no limitations were set on language, gender, number of recruited patients in the reported trial, or place of study origin. In addition, the Boolean operators (AND, OR, NOT) were also incorporated at relevant places in the search engines to both narrow and widen the range of results. The published titles encountered were carefully reviewed and assessed for potential inclusion or exclusion from the study. Furthermore, the references from selected studies were utilized as an additional search strategy to find extra trials (Figure 1).

Eligibility criteria

All RCTs had to compare the risk of post-operative SSIs in patients undergoing laparotomy for extended types of general, colorectal, upper gastrointestinal, hepato-biliary, or pancreatic surgery. Included RCTs should have compared laparotomy wound irrigation by PVI versus NS.

Data acquirement

Data reported in the encompassed RCTs were acquired by two distinct authors on a predefined meta-analysis data extraction form. The item was compared and discovered to have a reasonable level of agreement among the reviewers. The procured data consisted of a list of authors, title of the published study, journal of publication, country and year of the publication, testing sample size (with sex differentiation when applicable), the number of patients in each group, treatment protocol for each intervention, post-operative SSIs, and duration of follow up. Following final data extraction, a meticulous discussion took part between the independent reviewers and, if any contrasts were found, a mutual agreement was negotiated.

Evidence procurement

For statistical exploration and calculations, the software package RevMan provided by the Cochrane Collaboration (London, UK) was used [19,20]. The odds ratio (OR) with a 95% confidence interval (CI) was implemented to outline the combined outcome for binary data on the incidence of post-operative SSIs. The calibration of the combined outcome was made possible by applying the random-effects model [21,22]. Heterogeneity among included studies was explored using the chi^2^ test, with significance set at p < 0.05, and was quantified [23] using the I2 test with a maximum value of 30% identifying low heterogeneity [23], 30%-60% identifying a moderate heterogeneity, and more than 90% set as significantly high heterogeneity. The Mantel-Haenszel method was used for calculating OR under the random effect model analysis [24]. In a sensitivity analysis, 0.5 was added to each cell frequency for trials in which an event had taken a course in neither the treatment nor control group, according to the recommended method by Deeks et al. [25]. The Cochrane Collaboration calculation guidelines were resorted to in events where the standard deviation was not obtainable [21]. This involved assumptions that both groups had the same variance, which may not be factual, and variance was estimated from either the range or p-value. The pooled estimated difference was calculated based on each trial estimate variance's effect weights. A forest plot was used for the graphical exhibition of the results. The square around the estimate signified the accuracy of the estimation (sample size), while the horizontal line represented the 95% CI. The methodological quality of the included trials underwent evaluation in line with the standards of the Cochrane Collaboration in addition to the guidelines of Jaddad et al., Chalmers et al., and Rangel et al. [26-28].

Endpoint

The prime endpoint in this meta-analysis comparing laparotomy wound irrigation by PVI vs. NS in abdominal surgery patients was reached by examining the post-operative SSIs.

Outcome

Standard medical databases search generated 78 potential studies. Supplemental sources such as reviewing the bibliography of the published trials also contributed to three further studies that could be included in this review. The titles and abstracts of 78 studies were skimmed, and 55 studies were considered irrelevant. The remaining 23 studies were thoroughly examined and brought down to 13 studies that proved eligible to be included in the systematic review (Figure 1).

Quantitative Involved Trials Review

The 13 RCTs [29-41] involving 3816 patients fulfilled the inclusion criteria for the meta-analysis conducted per the principles of the Cochrane Collaboration. The PRISMA flow chart in trial search, deletion and selection, and study inclusion is presented in Figure 1. Although the inclusion criteria in the reviewed trials slightly differed, laparotomy wound irrigation by PVI and subsequent SSI condition as the primary outcome was homogenous among all studies. The number of recruited patients in the trials varied from 116 [33] to 941 [36] and the weighted percentage contributed by each trial to generate a summated evidence varied from 0.16% [34] to 2.87% [33]. Culminated evidence derived from the database search displayed that trials on this particular matter were being conducted for at least 43 years, spanning from 1979 [39] to 2022 [36], leading us to believe in the ongoing interest in finding the role of PVI in SSIs prevention. Follow-up duration for the included RCTs wavered between seven and 120 days. The panel of recruited patients extended beyond individuals undergoing laparotomy for general, colorectal, upper gastrointestinal, hepato-biliary, and pancreatic surgery, to ones with pelvic, gynecological, and oncological indications.

Qualitative Involved Trials Review

The methodological quality of the included trials was determined by covering quality indicators, namely, randomization technique, blinding, concealment, power calculations, intention-to-treat analysis, trial registration, and ethics committee approval. These indicators were evaluated by using the GRADEpro tool and other tools [26-28] and summary evidence was generated (Table 1).

**Table 1:** GRADEpro summary of evidence. Patient or population: Patients attempted for surgical site infection (SSI) prevention. Intervention: Povidone-iodine laparotomy wound irrigation. Comparison: Saline. Grade working group grades of evidence:- High quality: Further research is very unlikely to change our confidence in the estimate of effect. Moderate quality: Further research is likely to have an important impact on our confidence in the estimate of effect and may change the estimate. Low quality: further research is very likely to have an important impact on our confidence in the estimate of effect and is likely to change the estimate. Very low quality: We are very uncertain about the estimate. * The basis of the assumed risk (e.g., the median control group risk across studies) is provided in the footnotes. The corresponding risk (and its 95% CI) is based on the assumed risk in the comparison group and the relative effect of the intervention (and its 95% CI).

Povidone-iodine laparotomy wound irrigation compared to saline for prevention of surgical site infection						
Outcomes	Illustrative comparative risks* (95% CI)		Relative effect (95% CI)	No. of participants (studies)	Quality of the evidence (grade)	Comments
	Assumed risk	Corresponding risk				
	Saline	Povidone iodine laparotomy wound irrigation				
SSI odds ratio. Follow-up: mean six weeks	Study population		OR = 0.54 (0.3 to 0.98)	3816 (13 studies)	High quality	-
	79 per 1000	44 per 1000 (25 to 78)				
	Moderate					
		46 per 1000 (26 to 80)				

Analysis of the Primary Outcome Measure

**Figure 2:**
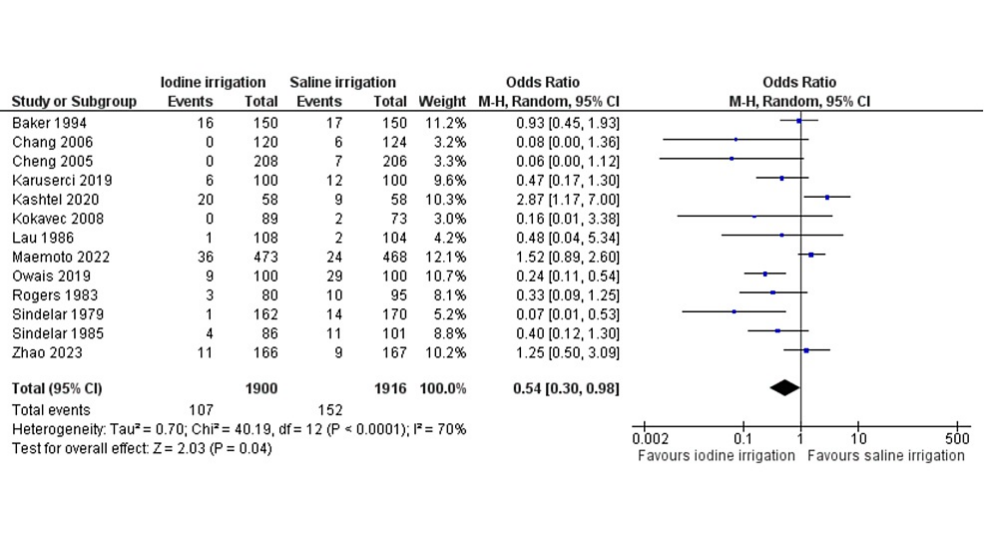
Forest plot showing the incidence of surgical site infections. The odds ratios are shown with 95% confidence intervals. Baker et al. (1994) [29], Chang et al. (2006) [30], Cheng et al. (2005) [31], Karuserci et al. (2019) [32], Kashtel et al. (2020) [33], Kokavec et al. (2008) [34], Lau et al. (1986) [35], Maemoto et al. (2023) [36], Owais et al. (2019) [37], Rogers et al. (1983) [38], Sindelar et al. (1979) [39], Sindelar et al. (1985) [40], Zhao et al. (2023) [41].

The individual OR and summated OR with 95% CIs analyzed using the random effects model meta-analysis of included RCTs are presented in Figure 2. Consequently, in the random effects model analysis, the incidence of SSIs was statistically lower in PVI (OR = 0.54, 95% CI (0.30, 0.98), Z = 2.03, p = 0.04). However, significant statistical heterogeneity (Tau^2^ = 0.70; chi^2^ = 40.19; df = 12; I2 = 70%; p = 0.0001) was also observed between the included trials.

Discussion

Evidence Briefing

Founded upon inspecting 13 RCTs on 3816 patients undergoing laparotomy for abundant reasons, PVI, as a wound irrigation solution, consolidated its advantage in reducing SSI risk. Hence, it would routinely be considered for laparotomy wound irrigation as a reducing measure of the functional and financial impact of SSI management.

Evidence Validity

The included studies report an assorted range of surgery types, which could be subdivided depending on laparotomy wounds into four categories: clean, clean-contaminated, contaminated, and dirty wounds. Therefore, concluding that the generated evidence is universally applicable is difficult. An appreciable number of trials focused on gynecological patients, impacting the generalizability of the results to all surgical patients. The number of recruited patients varied from 116 to 941, and each trial's contribution to the accumulated evidence ranged from 0.16% to 2.87%. The evidence indicates that trials on this subject have been conducted for at least 43 years, from 1979 to 2022. This highlights significant differences between participants and surgical techniques, while developing antibiotic resistance over time may also affect the results.

Evidence Quality

Based on the evaluation of the reported quality indicators and screening through the Cochrane tool for bias risk, and the GRADEpro tool (Table 1), the presented evidence may be labeled as adequate. However, on the background of study limitations, a degree of partiality was observed due to statistical heterogeneity besides methodological diversity seen in the inclusion criteria of included RCTs. As PVI wound irrigation is both cost-effective and time-saving, it could go without saying that its routine use may be recommenced.

Contrasting Reviews

Mueller et al. [17] reported a meta-analysis on 41 RCTs recruiting patients over 9000, which showed that intra-operative wound irrigation by any solution was associated with a reduced incidence of SSIs compared to no irrigation. Further subgroup analysis showed that the effect of reduced SSI incidence was strongest in colorectal surgery when wounds were irrigated with antibiotic solutions, in comparison with PVI or saline irrigation. However, according to the quality assessment, all of the included cases were at notable bias risk. The current study evaluated the effect of PVI solely on all patients undergoing laparotomy for gastrointestinal and gynecological pathologies, and it is the largest study up to today, favoring the routine use of PVI wound irrigation to reduce SSIs incidence. De Jonge et al. [42] reported the data on 21 studies suggesting a low quality of evidence, demonstrating a statistically significant benefit of incisional wound irrigation with PVI in clean and clean-contaminated wounds, in addition to refuting the role of antibiotic irrigation in reducing SSIs. The conclusion of the study reported that low-quality evidence existed in using prophylactic incisional wound irrigation to prevent SSIs with an aqueous PVI solution, in addition to displaying insensible antibiotic irrigation use. This study reported a combined analysis of trials evaluating the role of wound irrigation with saline versus antibiotics solution resulting in misleading outcomes. The current study reports the evaluation of PVI's prophylactic role in reducing SSIs, which has demonstrated superiority over normal saline or nothing. Fu et al. [43] reported a meta-analysis of 24 studies on 4967 subjects evaluating antibiotic solution irrigation, aqueous PVI, and saline irrigation. Again, it supported the use of PVI wound irrigation to reduce SSI incidence. The findings of our study are in concordance with the previously published three reviews.

Limitations

Several limitations of this study came into sight, particularly, the significant heterogeneity among included studies accounting for a degree of bias. It indicates diverse inclusion and exclusion criteria, and combined analysis of studies recruiting patients undergoing procedures with clean, clean-contaminated, and contaminated wounds resulting in outcomes with hard-to-overlook bias. Although this is the largest study recorded reporting the role of PVI-based laparotomy wound irrigation in reducing SSIs, the conclusion should still be interpreted cautiously due to these several limitations.

Implications

Putting RCTs evaluating the role of PVI in clean-contaminated or grossly contaminated into consideration is a must since this group presents with the highest incidence of SSIs. Equal consideration should be paid to trials focusing on upper gastrointestinal resections versus lower gastrointestinal resections to assess where PVI wound irrigation would be deemed beneficial. Likewise, the role of PVI wound irrigation after laparoscopic colorectal or gastric resections may also be explored.

## Conclusions

After conducting a thorough review of multiple research studies on suitable wound irrigation solutions for laparotomy surgeries over the past few decades, it has been observed that PVI is the most effective in reducing the risk of SSIs. Additionally, using PVI has financial benefits as it helps limit expenses related to re-operation, and extended courses of antibiotics, and ultimately results in improved quality of life for patients.
